# Computational study on single molecular spectroscopy of tyrosin-glycine, tryptophane-glycine and glycine-tryptophane

**DOI:** 10.1038/s41598-017-16234-3

**Published:** 2017-11-20

**Authors:** Bing Yang, Shixue Liu, Zijing Lin

**Affiliations:** 1 Hefei National Laboratory for Physical Sciences at Microscale & CAS Key Laboratory of Strongly-Coupled Quantum Matter Physics, Department of Physics, University of Science and Technology of China, Hefei, 230026 China; 2 Department of Nanotechnology for Sustainable Energy, Kwansei Gakuin University, Gakuen 2-1, Sanda, Hyogo, 669-1337 Japan

## Abstract

Quantum chemistry calculations play a fundamental role in revealing the molecular structures observed in gas-phase spectroscopic measurements. The supersonic jet cooling widely used in single molecular spectroscopy experiment is a non-equilibrium process and often causes confusion on the theoretical and experimental comparison. A computational approach is proposed here to account for the effect of the non-equilibrium cooling on the experimental spectra and applied to the cases of tyrosin-glycine (YG), tryptophane-glycine (WG) and glycine-tryptophane (GW). The low energy conformers of YG, WG and GW are obtained through thorough conformational searches. The structural features and equilibrium distributions of conformations and the energy barriers for conformer conversions are then determined. Three classes of transition energy barriers, high, medium and low, are found for the conversions among conformers with distinctly different, similar and the same structural types, respectively. The final conformation populations are determined by assuming an initial temperature of about 450 K and allowing for only the conformation conversion with a low energy barrier to occur during the rapid cooling process. The results provide a natural explanation for the numbers of YG, WG and GW conformations observed experimentally. The theoretical conformation assignments are also in good agreement with the experimental IR data.

## Introduction

The three-dimensional structure of biomolecule is a basic factor determining its biological functions and properties such as molecular recognition and ligand bindings. Much experimental and theoretical effort has been devoted to elucidate the biomolecular structure and the interactions therein. Single molecular spectroscopy techniques have been frequently used to probe the structures of biomolecules ranging in size from simple amino acids to entire protein assemblies^1–5^. However, the spectroscopic experiment yields only indirect structural information. The molecular structures can be determined only by comparing the experimental spectra with the results of quantum chemistry calculations^6–9^. Unfortunately, though the spectroscopic technique and high accuracy quantum chemistry computations have reached some level of maturity, the conformation assignment is still encountered with ambiguity^7,8,10^.

The practice of comparing the theoretical and experimental spectra often suffers some of the shortcomings: (1) Theoretical IR spectra are computed based on a set of candidate structures determined by some crude conformational search^7,10^. As the set of candidate structures may miss important low energy conformations of the molecule, the quality of structural assignment thus determined is rather questionable. (2) The structural assignment is made based on the best match between the theoretical and experimental spectra. However, the matched structures may be unfavorable based on the consideration of conformational energies^1–3^. (3) It is quite common to see the absence of the theoretically predicted low energy structures in the experimental spectra^9^. To explain the experimental results, some non-equilibrium dynamic mechanism is required. For example, a mechanism of non-radiative deactivation of excited states of folded peptide conformers has been proposed^11,12^. The non-radiative deactivation mechanism is capable of explaining a number of experimental spectra and is gaining an increased acceptance^11–19^. However, the facts about the absence of folded WG conformer and the presence of folded GW conformer in the experiments cast serious doubt on the non-radiative deactivation mechanism as the mechanism should be equally applicable to both cases of WG and GW (W = tryptophan, G = glycine)^8,10,18^. A more natural explanation is desirable. (4) The effects of the supersonic expansion cooling process on the experimental spectra are considered only vaguely. As the global free energy minima of WG and WGG at 300 K match the experimental results^9^, it is suggested that the high population structures at about 300 K might have approximately been kept during the supersonic cooling process^20^. Similar result is also found recently on the IR spectra of YG conformers (Y = tyrosine)^21^. However, the number of conformations predicted by the free energy consideration is substantially more than that observed experimentally. It is then natural to conclude that the experimentally observed structures are the combined effect of conformational distribution at some high formation temperature and the dynamics of non-equilibrium cooling process^20–22^. Unfortunately, no serious effort is made to establish the correspondence between the computationally determined and experimentally observed structures.

This paper reports a detailed theoretical study on the conformations of YG, WG and GW, with the aim of providing an improved explanation of the experimental results^7,8,10^. The low energy structures are determined through extensive conformational searches and characterized based on their structural features. The conformational transition energy barriers within each structural class and between different structural classes are computed. The initial conformation distributions and the transition energy barriers are used to predict the detectable structures after the rapid supersonic expansion cooling process. The analysis provides a clear and coherent mechanism that agrees well with the experimental observations. The computational approach described here is a reliable way for determining the structures observed by supersonic jet cooling based molecular spectroscopy.

## Results and Discussion

### Tyrosin-glycine

#### Conformational search results

**Figure 1:**
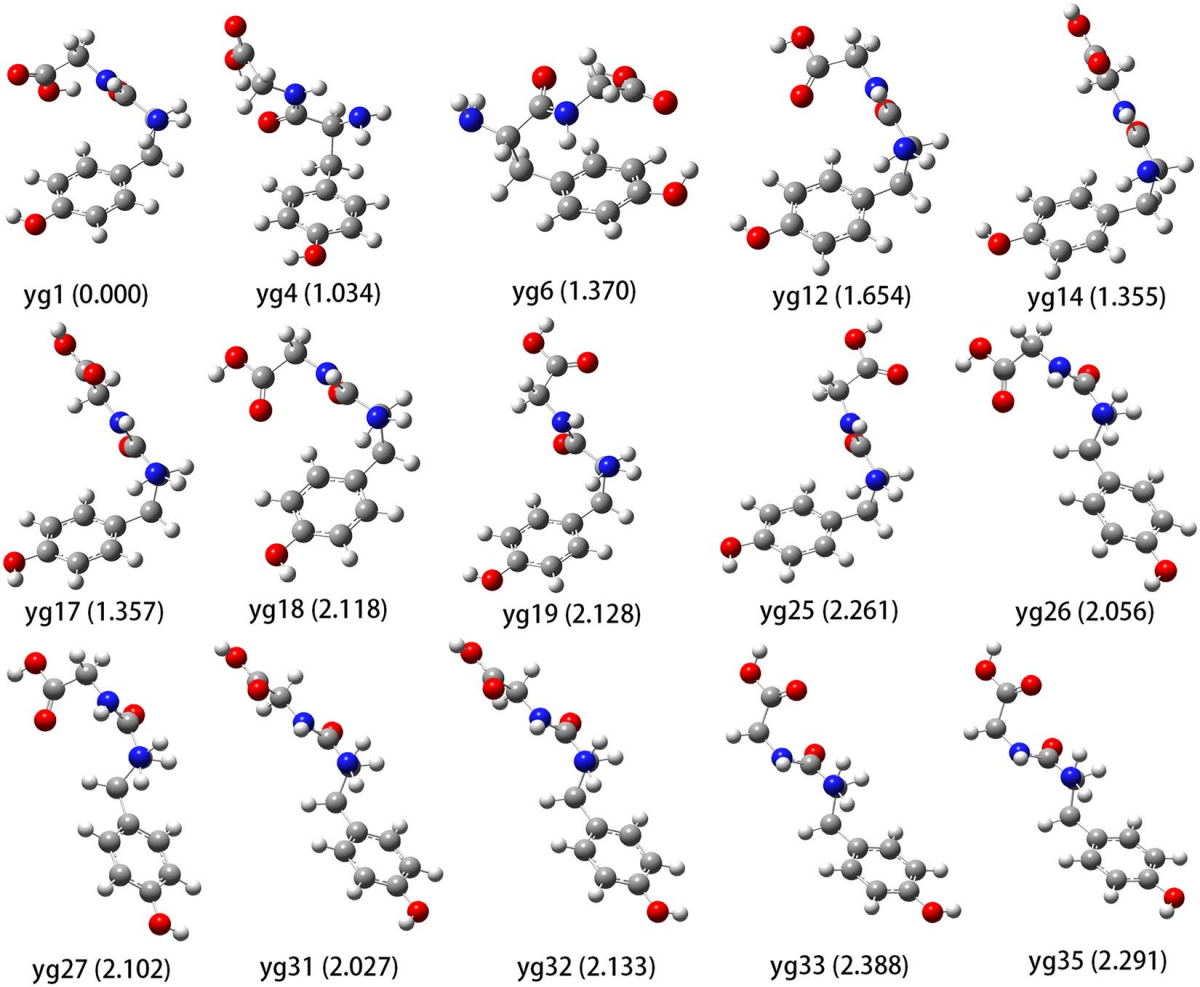
Representative YG conformations. Relative total energies (the sum of the electronic energy and the zero-point vibrational energy, in kcal/mol) of the conformers are shown in the parentheses.

Through extensive conformational searches, many local minima were found on the potential energy surface. Table 1 shows the relative energies, hydrogen bonds (H-bonds), structural types and equilibrium distributions of YG conformers of interest. The structures of representative conformers are displayed in Fig. 1. Here, a YG conformer is denoted as yg*n* and the numeral suffix n refers to its position in the conformational sequence ordered by ascending electronic energies. An H-bond is defined by a cutoff distance of 2.8 Ǻ.

**Table 1:** Relative electronic energies (Energy, in kcal/mol), H-bond networks and structural types (Type) for all YG conformers (Conf.) of interest. The equilibrium distributions (%) at three representative temperatures are also shown but an equilibrium content below 1% is denoted as “—”.

Conf.	Energy	H-bonds^1*^		Type^2*^	Distributions		
		Backbone	Main/Side-Chain		98K	298K	450K
yg1	0.000	N_PB_H···N_1_; O_T_H···OC_PB_	N_1_H···π	A1-γ_D_(F)-g+/+	100	11.42	3.05
yg2	0.523	N_PB_H···N_1_; O_T_H···OC_PB_	O_S_H···O = C_T_	A2-γ_D_(F)-g+/+	—	—	—
yg3	0.786	N_PB_H···N_1_; O_T_H···OC_PB_	N_1_H···π	A1-γ_D_(F)-g+/−	—	4.25	1.72
yg4	0.973	N_PB_H···N_1_; O_T_H···OC_PB_	N_1_H···π	A1-γ_L_(F)-g+/+	—	3.18	1.56
yg5	1.121	N_PB_H···N_1_; O_T_H···OC_PB_	N_1_H···π	A1-γ_L_(F)-g+/−	—	2.98	1.57
yg6	1.230	N_1_H···OC_PB_; O_T_H···OC_PB_	O_S_H···O = C_T_	B-γ_L_(F)-a/-	—	—	—
yg7	1.779	N_PB_H···N_1_; O_T_H···OC_PB_	N_1_H···π	A2-γ_D_(F)-g−/−	—	3.17	2.34
yg8	1.783	N_PB_H···N_1_; O_T_H···OC_PB_	N_1_H···π	A2-γ_D_(F)-g−/+	—	3.54	2.53
yg9	1.819	N_PB_H···N_1_; O_T_H···OC_PB_	N_1_H···π	A2-γ_L_(F)-g−/−	—	1.59	1.24
yg10	1.843	N_PB_H···N_1_; O_T_H···OC_PB_	N_1_H···π	A2-γ_L_(F)-g−/+	—	2.01	1.55
yg11	1.858	N_PB_H···N_1_; O_T_H···OC_PB_	—	A2-γ_L_(F)-g+/+	—	2.03	1.45
yg12	1.880	N_PB_H···N_1_	N_1_H···π	A1-ε_D_(E)-g+/+	—	2.25	1.88
yg13	2.001	N_PB_H···N_1_; O_T_H···OC_PB_	—	A2-γ_L_(F)-g+/−	—	1.35	1.07
yg14	2.011	N_PB_H···N_1_	N_1_H···π	A1-β(E)-g+/+	—	11.62	8.65
yg17	2.113	N_PB_H···N_1_	N_1_H···π	A1-β(E)-g+/−	—	10.57	8.10
yg18	2.360	N_PB_H···N_1_	N_1_H···π	A1-ε_D_(E)-g+/−	—	1.02	1.10
yg19	2.375	N_PB_H···N_1_	N_1_H···π	A1-ε_L_(E)-g+/+	—	1.49	1.58
yg25	2.552	N_PB_H···N_1_	N_1_H···π	A1-ε_L_(E)-g+/−	—	1.33	1.55
yg26	2.613	N_PB_H···N_1_	N_1_H···π	A2-ε_D_(E)-g−/+	—	3.37	3.72
yg27	2.619	N_PB_H···N_1_	N_1_H···π	A2-ε_D_(E)-g−/−	—	3.91	4.44
yg29	2.710	N_PB_H···N_1_; O_T_H···OC_PB_	N_1_H···π	A1-γ_D_(F)-g−/+	—	—	1.00
yg30	2.721	N_PB_H···N_1_; O_T_H···OC_PB_	N_1_H···π	A1-γ_D_(F)-g−/−	—	—	1.00
yg31	2.723	N_PB_H···N_1_	N_1_H···π	A2-β(E)-g−/+	—	4.35	4.99
yg32	2.725	N_PB_H···N_1_	N_1_H···π	A2-β(E)-g−/−	—	2.75	3.23
yg33	2.744	N_PB_H···N_1_	N_1_H···π	A2-ε_L_(E)-g−/−	—	2.18	2.87
yg35	2.777	N_PB_H···N_1_	N_1_H···π	A2-ε_L_(E)-g−/+	—	3.07	3.93
yg38	2.905	N_PB_H···N_1_; O_T_H···OC_PB_	N_1_H···π	A1-γ_L_(F)-g−/+	—	—	1.15
yg51	3.489	N_PB_H···N_1_	N_1_H···π	A1-ε_D_(E)-g−/−	—	1.17	2.02
yg52	3.493	N_PB_H···N_1_	N_1_H···π	A1-ε_D_(E)-g−/+	—	1.17	2.07
yg56	3.607	N_PB_H···N_1_	N_1_H···π	A1-β(E)-g−/−	—	—	1.56
yg57	3.638	N_PB_H···N_1_	N_1_H···π	A1-β(E)-g−/+	—	—	1.51
yg58	3.650	N_PB_H···N_1_	—	A2-ε_L_(E)-g+/+	—	1.00	1.90
yg63	3.900	N_PB_H···N_1_	N_1_H···π	A1-ε_L_(E)-g−/−	—	—	1.53
yg67	3.958	N_PB_H···N_1_	N_1_H···π	A1-ε_L_(E)-g−/+	—	—	1.66
yg71	4.183	N_PB_H···N_1_	N_1_H···π	A2-α_L_(E)-g−/−	—	—	1.29
yg72	4.214	N_PB_H···N_1_	N_1_H···π	A2-α_L_(E)-g−/+	—	—	1.04
yg100	5.416	N_1_H···OC_PB_	N_1_H···π	B-β(E)-g−/−	—	—	1.15

1*: N_1_ refers to the N-terminal nitrogen atom, N_PB_ is the nitrogen atom in the peptide-bond, and O_T_ stands for the oxygen atom of the C-terminal hydroxyl.

**Figure 2:**
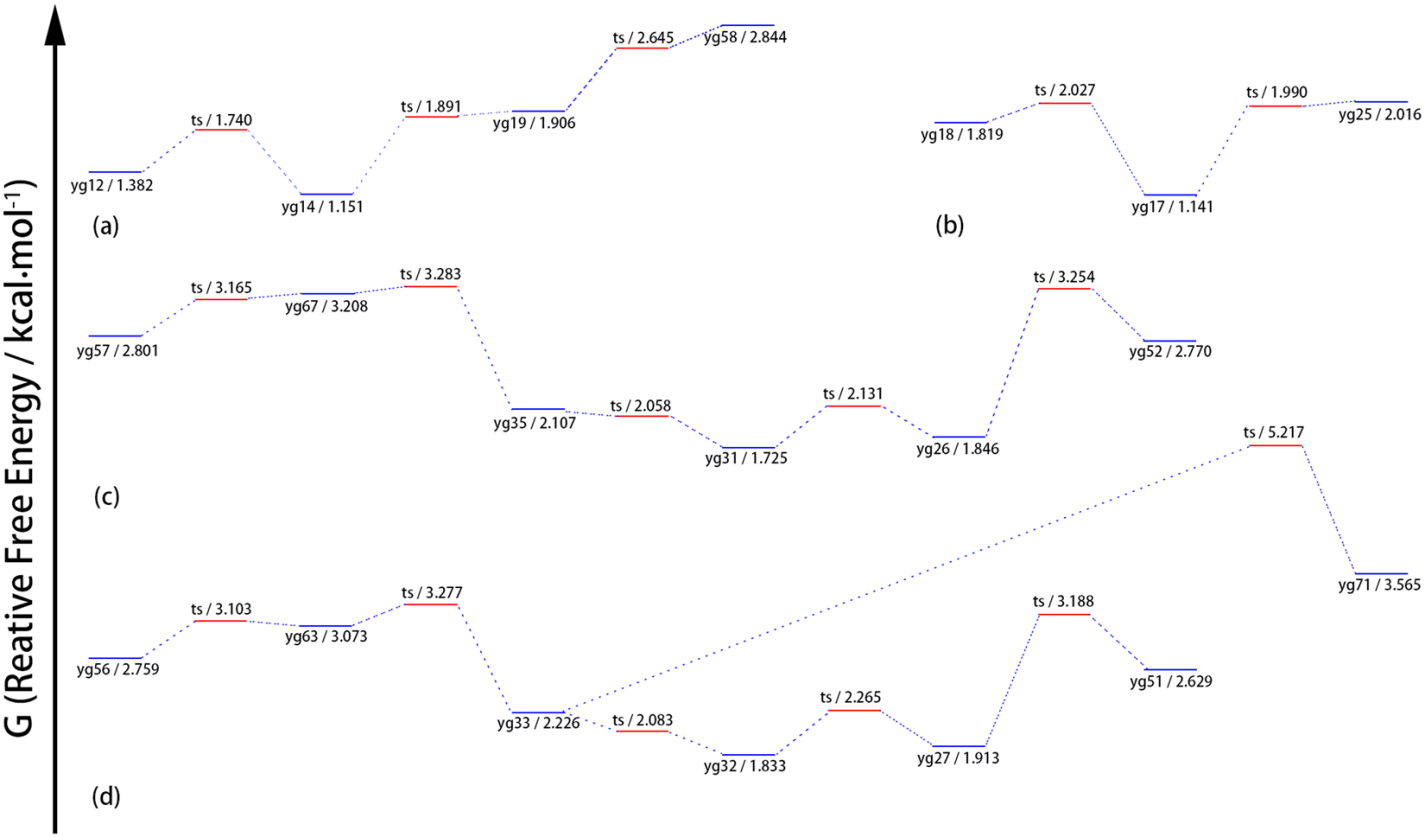
The free energy profile for four groups of YG conformers with extended backbone structures: (**a**) conformers with g+/+ side chains, (**b**) conformers with g+/− side chains, (**c**) conformers with g−/+ side chains, (**d**) conformers with g−/− side chains. “ts” in the graph stands for “transition state”.

2*: The label of structural type consists of four parts. The first part, A1, A2 or B, is used to indicate the swing direction and H-bond type of the amino terminus. See yg14, yg31 and yg6 in Fig. 2 for the configurations of A1, A2 and B, respectively. The second part of the label refers to the dihedral angle (ϕ_2_, φ_2_) in the Ramachandran plot. The third part of the label indicates the swing direction of the side chain, with *g*+, *g*−and *a* correspond to the dihedral angle of N−C_α_-C_β_-C_γ_ close to +180, +60, and −60°, respectively. The last part, + or − after the slash, denotes the side chain hydroxyl orientation.

As shown in Table 1 as well as Fig. 1, most of the low energy conformers adopt folded configurations (γ type backbone), associated with the formations of strong H-bonds. However, the conformational distribution shows a strong temperature dependence or entropic effect. With the increase of temperature, the extended structures, typically with a β or ε backbone and a g- side chain, become increasingly more important in the conformation ensemble. Only one H-bond concerning the peptide bond is formed in the extended α/β/ε backbone. Moreover, the g- orientation is helpful to keep the side chain away from the C-terminal backbone and allow for a more liberal swing of the side chain to increase the entropic effect. As a result of the entropic effect, the equilibrium ensemble, though dominated by the low-energy folded structures at low temperature, consists of mainly extended structures at the room temperature or above.

As seen in Table 1, the energy of a conformer of interest can be more than 5 kcal/mol above the global minimum. At T~450 K, the relative electronic energies for the most populous structures fall into the interval of 2 to 3 kcal/mol. Notice that T~450 K is often expected for the relevant experimental conditions such as laser heating^23,24^. Therefore, conformers within a sufficiently large energy range of the global minimum should be thoroughly searched in order to reliably determine the most important conformers under the experimental condition.

#### Interpretation of the experimental results

Four different YG conformers were observed by double-resonance spectroscopic technique^7^. The experimental results were obtained after rapid cooling by supersonic expansion that resulted in a very low temperature (about 10 K). As shown in Table 1, there is only one single structure in the equilibrium conformational ensemble at 10 K. The discrepancy between the experimental results and the theoretical equilibrium conformational distribution is not surprising, however. The discrepancy is caused by the fact that the conformational ensemble does not have the time to reach its equilibrium state in the rapid cooling process. Instead, a conformer existed before the supersonic expansion may only relax to a local minimum connected by a low energy barrier in the supersonic expansion cooling process^6^. Therefore, a proper explanation of the experimental results requires a detailed examination of the dynamics of the rapid cooling process. The theoretical and experimental comparison is further complicated by uncertainties regarding the conformational ensemble before the supersonic expansion cooling. The initial conformational ensemble was obtained by laser ablation and may not reach a thermodynamic equilibrium before the cooling. Consequently, a rigorous account of the experimental results may require a complete modeling of the laser heating and jet cooling processes that is rather difficult and not attempted here. Instead, it is illustrated below that the experiment can be accounted for quantitatively by using an effective laser heating temperature and considering the effect of transition energy barriers on the conformational conversions in the rapid cooling process.

The free energy barriers for transitions among conformers of similar and different structural characteristics were examined systematically. The free energy barriers are found to be weakly temperature dependent and generally decrease with the decease of temperature. However, the free energy barriers at low temperature (10 K) are found to be representative and are used in the following discussion.

The obtained energy barriers can be classified into three categories: high, medium and low. The high energy barrier (≥8 kcal/mol) corresponds to the transformation between conformers with very different backbone configurations. For example, the transition of yg4↔yg1 corresponds to the transition of γ_L_ ↔ γ_D_ in the folded backbone and the transition of yg12/yg14↔yg1 corresponds to the transition of ε/β in the extend backbone ↔ γ in the folded backbone. The energy barrier for such a transformation is high as the transition process involves the breakage and reformation of strong H-bond. This kind of transformation requires a very long relaxation time to occur and may be safely ignored under the experimental condition.

The medium energy barrier (2~8 kcal/mol) is associated with the transformation between conformers with different side chain swing directions. Examples include the transition of yg12↔yg52 for the side chain transformation of g+ ↔ g− and yg14 ↔ yg17 for the transformation of the side chain hydroxyl orientation of + ↔ −. Though no H-bond breaking is involved, the energy barrier is moderately high as the transformation is encountered with some steric hindrance. The transformation with a medium energy barrier is also expected to be negligible during the rapid cooling process of supersonic expansion^6^.

A low energy barrier (≤2 kcal/mol) is found for the transition involving only the rotation of terminal groups, e.g., yg58↔yg36 (the swing of amidogen direction), or conformers with similar extended backbone configurations, e.g., yg28↔yg36 (β↔ε of the extend backbone). The transformation between conformers separated by the low energy barrier is expected to be sufficiently fast and can easily take place during the supersonic expansion cooling process.

Information about the effective temperature of the conformational ensemble obtained by laser-induced desorption is required to understand the conformational distribution after the supersonic expansion cooling. Based on thermal equilibrium desorption kinetics, the temperature is found to be in the range of 380–450 K or 550–670 K, depending on the heating condition^23^. For convenience, an effective temperature of 450 K is temporarily assumed for the conformational ensemble before the supersonic expansion. The use of 450 K is arguable due to a lack of firm experimental support. However, the choice is found to produce results in good agreement with the experiment on YG as well as WG and GW, as to be seen below. Moreover, it is noted that the following discussion is not qualitatively affected by allowing for an uncertainty of 50 K in the assumed temperature.

With the assumed temperature of 450 K, Table 1 shows that many conformers, including the folded conformers of yg1~yg11, yg13, yg29, yg30, yg38 and yg100, are present in the equilibrium ensemble. However, as discussed above on the energy barriers, no conversion between a folded conformer and an extended conformer may occur. Similarly, there is no conversion between differently folded conformers. As a result, no folded conformer, including yg1, may accumulate through the conversion process to have a population above 5%.

The conversion between extended conformers with a similar side chain configuration can take place easily. There are four types of side chain configurations, g+/+, g+/−, g−/+ and g−/−. Among the conformers of interest, yg12, yg14, yg19 and yg58 belong to g+/+, yg17, yg18 and yg25 belong to g+/−, yg6, yg31, yg35, yg52, yg57 and yg67 belong to g−/+, while yg27, yg32, yg33, yg51, yg56, yg63 and yg71 belong to g−/−. The four groups of conformers are separated by some medium energy barriers and the inter-group conversion is negligible. However, the energy barrier for intra-group conformational conversion is quite low. Figure 2 shows the free energy profile for the four groups of conformers. As shown in Fig. 2a, the energy barriers for the conversions of g+/+conformers are all less than 0.4 kcal/mol. The four conformers would converge to their local free energy minimum, yg14, in the cooling process. As a result, yg14 has a 14% concentration in the final conformational ensemble. Similarly, there are about 11% of yg17, 18% of yg31 and 17% of yg32 in the final conformational ensemble. The final concentrations of yg14, yg17, yg31 and yg32 are all above 10% and much higher than that of any other conformers. The four conformers should therefore be observable, while others may be indistinguishable from the background noise. The theoretical analysis is in good accord with the experimental findings.

**Figure 3:**
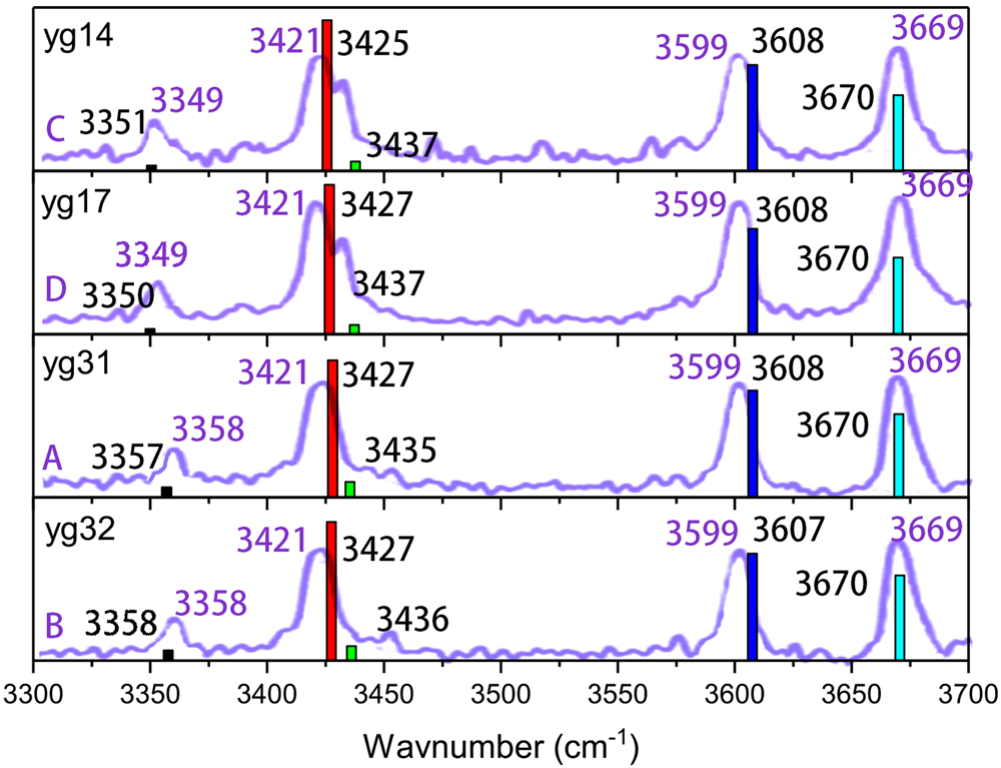
Comparison of the experimental (curves and numbers in purple) and theoretical (colored bars and numbers in black) IR spectra of YG conformations. The experimental results are taken from ref.^7^.

To further validate the above conformational analysis, IR spectra of yg14, yg17, yg31 and yg32 are computed and compared with the experiment. For the comparison, the theoretical harmonic frequencies should be scaled to account for the anharmonic effect. As the scaling factor for M062X/6-311++G** is unclear while that for B3LYP/6-31G** is well documented, the four conformers are re-optimized at the B3LYP/6-31G** level and the resulting frequencies are scaled by a factor of 0.9602 as recommended in literature^7^. Figure 3 shows the comparison of theoretical and experimental IR spectra. Clearly, the theoretical characteristic IR frequencies are in excellent agreement with the experiment, providing a strong support of our conformational assignment.

It is therefore concluded that the experimentally observed structures are resulted from the dynamic process of relaxing the conformations obtained with laser heating to local minima connected with low energy barriers. It is possible that the observed structures do not correspond to the global minimum on the FES of any temperature. In particular, the folded low energy conformers, including the final global minimum, are not detected due to unfavorable entropic effect and the energy barrier prohibited structural conversion. There is no need to invoke the non-radiative deactivation mechanism to explain the missing of the global minimum in the experimental spectra. Besides, the puzzle of finding only four instead of many more conformers is solved by the conversions of conformers connected with low energy barriers.

### Tryptophane-glycine

Similar to YG, the relative electronic energies, H-bonds, structural types and equilibrium distributions of WG conformers of interest are determined. Details are referred to Table 1S of the Supplementary Information (SI). The structures of representative conformers are displayed in Fig. 1S of SI. Like the case for YG, the global energy minimum of WG, wg1, adopts a folded backbone with a structural type of A1-γ_D_(F)-g+/+. Similarly, the equilibrium population of WG conformers also shows a strong entropic effect and the extended structures increase their importance with the increase of temperature. Very different from the case of YG, however, wg1 remains to be the most populous conformer at 450 K, with a concentration of 9.9%. Naturally, the chance of detecting the folded conformation of wg1 is rather high.

**Figure 4:**
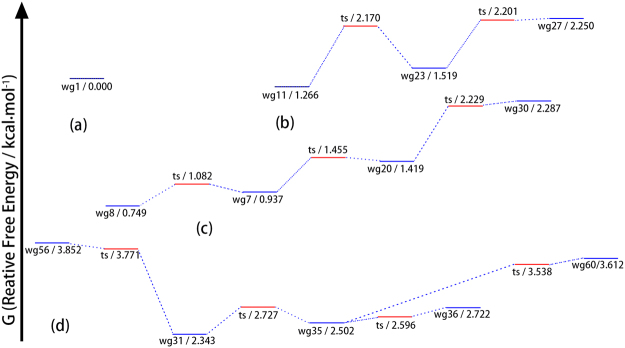
The free energy profile for four groups of WG conformers: (**a**) folded backbone conformer, (**b**) extended backbone conformers with g+/+ side chain, (**c**) extended backbone conformers with g+/− side chains, (**d**) extended backbone conformers with g−/+ side chains.

The potential energy surface of WG is characteristically the same as that of YG regarding the energy barriers for conformer transformations. Figure 4 shows the free energy profiles for four sets of WG conformers that are likely to be observed. After the supersonic jet cooling, the expected concentrations of wg1 (folded backbone), wg8 (extended backbone with g+/− side chain), wg11 (g+/+) and wg31 (g−/+) are 9.9%, 10%, 15% and 16%, respectively. The theoretical results of four high population conformers agree well with the double resonance experiment that yields four observed structures^8,10^. The agreement strongly suggests that the folded structure of wg1 is long lived in the conformational ensemble and detected experimentally. That is, even though wg1 exhibits a folded backbone and a backbone-side chain NH-π dispersive interaction, wg1 is not subjected to the non-radiative deactivation mechanism.

It should be pointed out that the g−/− conformation, wg43, wg46 and wg48, has a total population of only 5% and may not be observable due to low signal intensity. In comparison, the concentration of the g−/− conformation of YG, yg32, is as high as 17%. That is, although the overall structural types of YG and WG and the associated transition energy barriers are characteristically the same, the observable structures are different. It is necessary to conduct a detailed computational study dedicated to the interested molecule in order to provide a reliable explanation of the measurement results.

**Figure 5:**
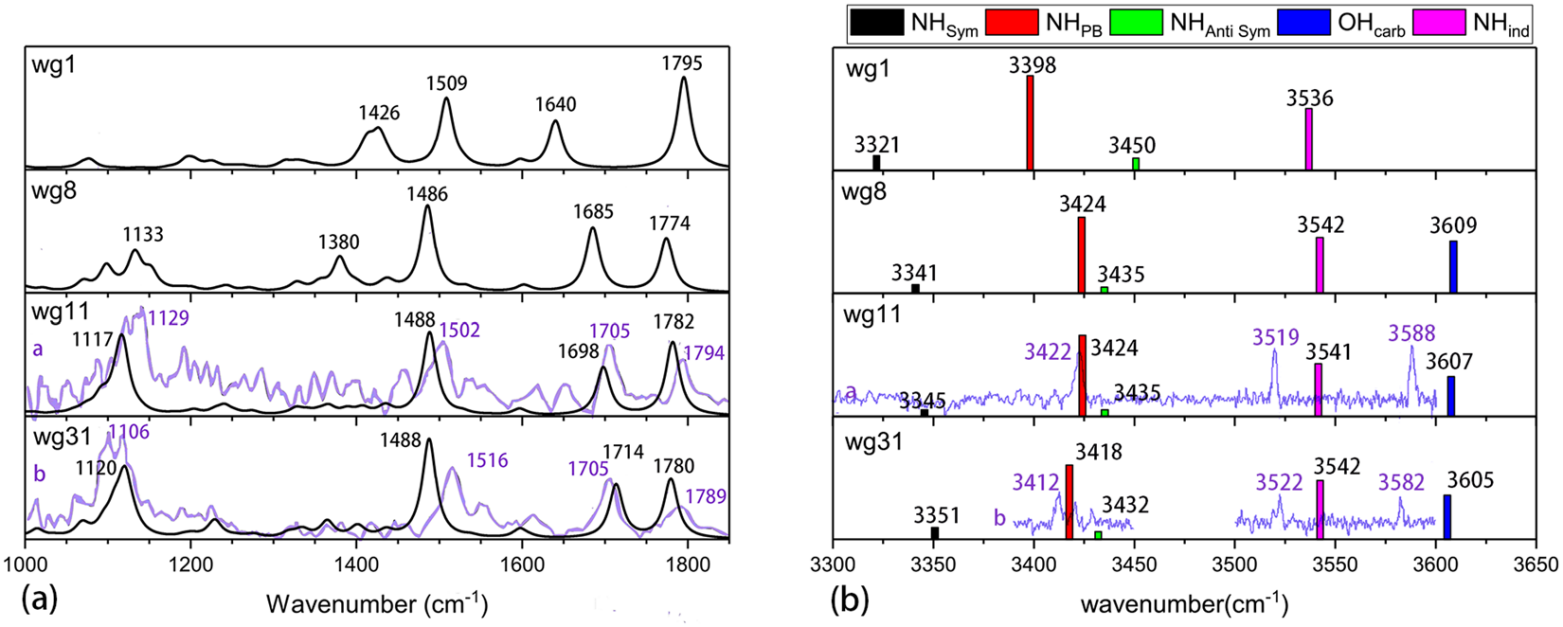
Comparison of the experimental (curves and numbers in purple) and theoretical (black curves and colored bars) observable IR spectra of WG conformations: (**a**) Frequency in the range of 1000 cm^−1^ to 1850cm^−1^ (the theoretical curves are Lorenzens with the full width at half maximum of 20 cm^−1^); (**b**) Frequency in the range of 3300 cm^−1^ to 3700 cm^−1^. The experimental results are taken from refs^8,10^.

Figure 5 shows the comparison of the experimental and theoretically observable IR spectra of WG conformations. Considering that four WG conformers are identified in the R2PI and UV-UV hole-burning experiments^10^, the IR spectra of wg1 and wg8 missed in the experiment are also shown as they may be useful for comparison with the future experiments. Notice, however, the IR spectrum of wg8 in the high frequency region has in fact been observed and the NH_pb_, NH_ind_ and OH frequencies are 3407, 3522 and 3598 cm^−1^, respectively^8,9^. As seen in Fig. 5, the agreement between the theoretical results and the available experimental data is quite satisfactory. The average and maximal deviations for seven characteristic vibrations are 14 and 28 cm^−1^, respectively. The comparison is based on a universal scaling factor, while the errors are smaller than the literature ones using vibration mode adjusted scaling factors^8,9^.

Based on the comparison shown in Fig. 5, the experimentally observed WG conformers *a*, *b*, *c* and *d* can be assigned respectively to wg11, wg31, wg1 and wg8 here^8,10^.

### Glycine-tryptophane

Table 2S of SI shows the relative electronic energies, H-bonds, structural types and equilibrium distributions of GW conformers of interest. The structures of representative conformers are displayed in Fig. 2S of SI. Once again, the global energy minimum of GW, gw1, adopts a folded backbone configuration. However, gw1 has a structural type of A2-γ_D_(F)-g−/−, instead of A1-γ_D_(F)-g+/+ for both yg1 and wg1. Besides, the extended conformer of gw3 has a total energy very close to that of gw1. Except for very low temperature, gw3 is the global free energy minimum due to favorable entropic effect.

**Figure 6:**
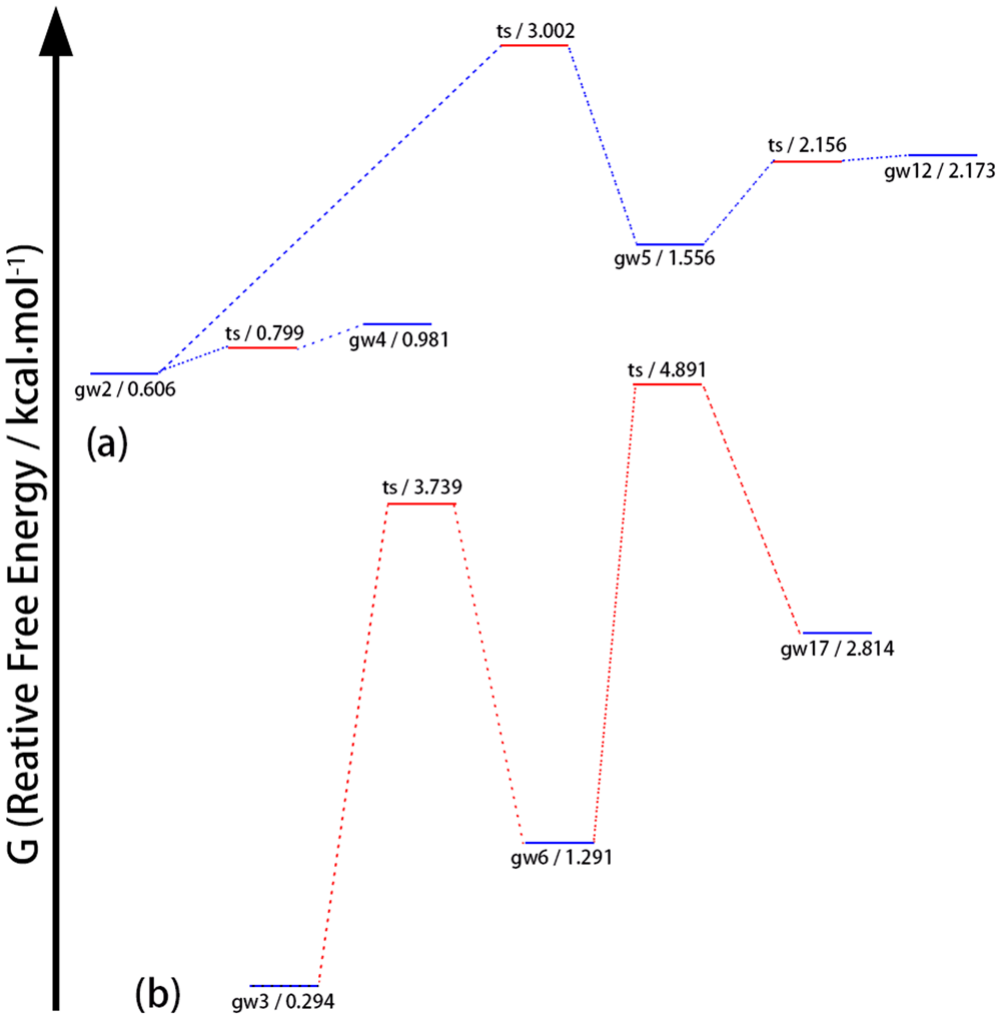
The free energy profile for two groups of GW conformers: (**a**) folded backbone conformers, (**b**) extended backbone conformers with g+/+ side chain.

Detailed analysis of the GW free energy profile has been carried out. It is noticed that the characterization about the energy barriers for conformer conversion discussed above, though remains overall correct, requires some minor revision. The free energy profiles for two sets of WG conformers of interest are illustrated in Fig. 6. On one hand, the energy barrier of converting gw5 with a g−/+ side chain to gw2 with a g+/+ side chain is only around 1.5 kcal/mol, as shown in Fig. 2S. The reduced energy barrier may be attributed to the presence of the N_PB_H···π H-bond. As a result of the reduced energy barrier, gw5 and gw12 may convert to gw2 in the jet cooling process. Based on the data shown in Table 2S, the final concentration of gw2 may reach above 20%. On the other hand, the energy barrier of converting gw6 to gw3, both with a g+/+ side chain, is increased to about 2.5 kcal/mol by the steric hindrance of the side chain. The conversion of gw6 and gw17 to gw3 may be limited. Nevertheless, gw3 has a high population at 18% by itself.

Considering the data shown in Table 2S, the conformational population for any one structural type other than that of gw2 and gw3 is less than 6%. Conformations with such low concentrations may be difficult to detect experimentally. That is, only gw2 and gw3, each with a concentration above 18%, can be easily observed. The conclusion is in good accord with the experimental findings^8,10^. Here again, the missing of gw1 is attributed to its low concentration in the conformational ensemble, instead of resulting from the non-radiative deactivation process. Besides, gw2 is predicted here to be observable even though its structural feature appears to be susceptible to the non-radiative deactivation mechanism^11,20^.

**Figure 7:**
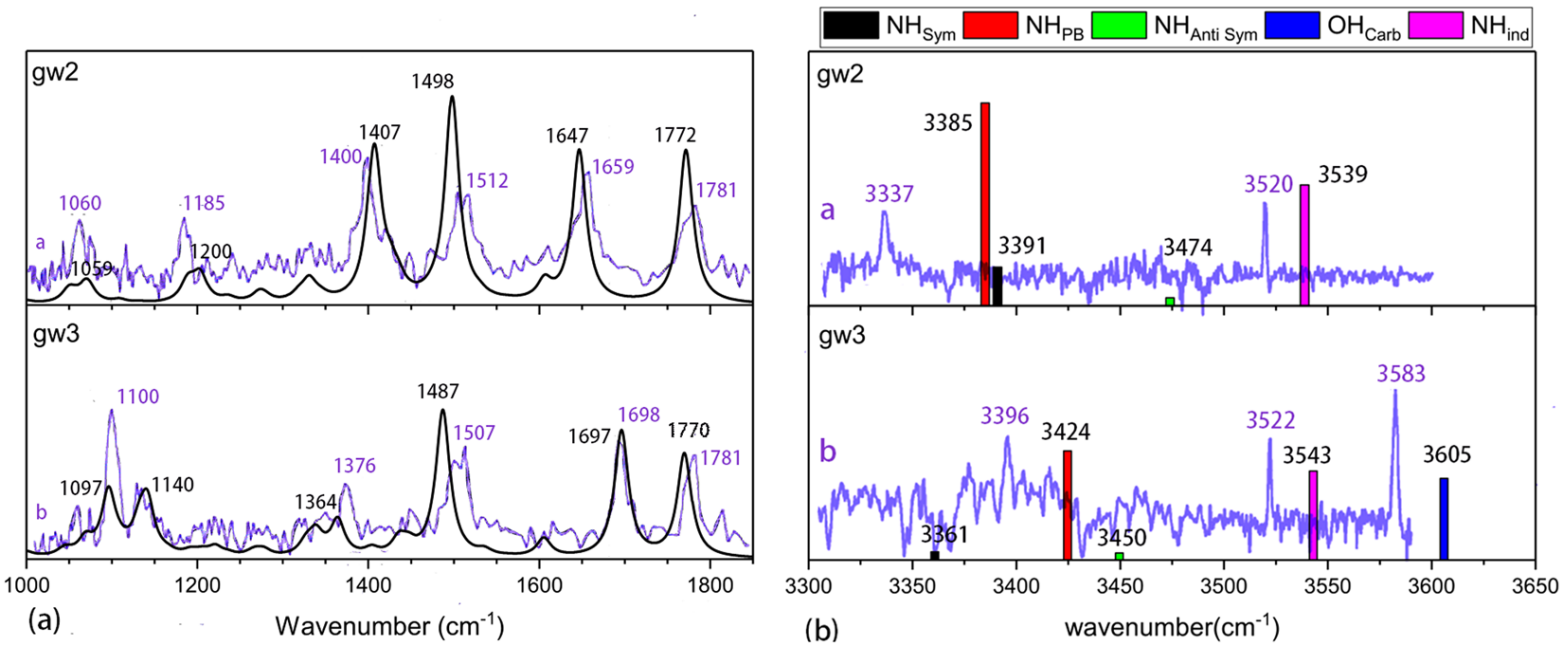
Comparison of the experimental and theoretical IR spectra of GW conformations: (**a**) mid-frequency region, (**b**) high-frequency region. The experimental results are taken from refs^8,10^. The theoretical results are obtained with B3LYP/6-31G** and scaled by a factor of 0.9602.

Figure 7 shows the comparison of the experimental and theoretically IR spectra of GW conformations. The agreement between the theory and experiment is quite good for the mid-frequency region. The difference is less than 10 cm^−1^ on average, while the biggest deviation is only 20 cm^−1^. The difference between the theory and experiment for the high-frequency region is much larger, however. On average, the difference is 28 cm^−1^. The largest deviation, 48 cm^−1^, is found for the N-H_PB_ vibration of gw2.

The deviation of 48 cm^−1^ appears somewhat large and causes some concern on the conformational assignment. The IR spectra of other probable conformers are computed. However, none of them is capable of producing an overall improved agreement with the experiment for the whole frequency range. A possible explanation is that, as known in the literatures^25,26^, the anharmonic effects for different vibrational modes are different and may be accounted for only through different scaling factors^25,26^. To explore other possible explanation, it is noticed that the backbone-side chain interactions in gw2 and gw3 (Fig. 2S) appear to be much stronger than that in yg14, yg17, yg31 and yg32 (Fig. 2) and in wg11 and wg31 (Fig. 1S). It is known that B3LYP is inadequate for describing strong H-π interaction, while the result by M062X is much more satisfactory^27,28^. Therefore, the IR spectra of gw2 and gw3 are recomputed with M062X/6-31G**. By requiring that M062X/6-31G** reproduces the average frequency for the six mid-frequency modes of gw2 identified experimentally, a scaling factor of 0.943 is deduced. With this scaling factor, the NH_PB_ and NH_ind_ frequencies of gw2 are found to be 3342 and 3504 cm^−1^, in comparison with the experimental values of 3337 and 3520 cm^−1^ (Fig. 7b), respectively. The theoretical NH_PB_, NH_ind_ and OH_carb_ frequencies of gw3 are 3382, 3515 and 3610 cm^−1^, while the experimental values are 3396, 3522 and 3583 cm^−1^, respectively. The average and largest differences between the theory and experiment for the high-frequency region are then reduced to 14 and 27 cm^−1^, respectively. Clearly, the agreement between the experiment and the theory is much improved by describing the H-π dispersion interaction with the M062X functional. Assigning the experimentally observed GW conformers *a* and *b* to gw2 and gw3^8,10^, respectively, is therefore validated.

## Conclusions

A computational framework for interpreting the results of supersonic jet cooling based single molecular spectroscopy is proposed. The method consists of the following steps: 1) a thorough conformational search to locate all low energy structures, 2) evaluating the equilibrium distributions of conformations at about 450 K, 3) classifying the structural features of low energy conformations, 4) determining the transition energy barriers for conformation conversions, 5) adding the populations of conformers with low energy barriers together to determine the contents of the relevant stable conformations after the supersonic expansion cooling. Highly populated conformations thus determined are the structures observed in the spectroscopic measurement.

As a rule of thumb, an energy barrier is deemed low if it is less than 2 kcal/mol. A conformer population is high and observable if it is above 8%. Moreover, a high energy barrier is expected for the conversion of conformers with different folded backbones or with extended and folded backbones. With inconsequential exceptions, a low energy barrier is found only for conformers with the same side-chain structural type. The empirical rules about the energy barriers can be used to avoid the computation extensive transition state calculations.

Each of the proposed computational steps has been considered before, but they have not been combined together yet in the interpretation of the single molecular spectroscopy. Here, the proposed computational approach as a whole is applied to the studies of the spectra of gaseous dipeptides YG, WG and GW. The results agree well with the experiments on the numbers of observable YG, WG and GW conformations. The theoretical conformation assignments are further validated by the available experimental IR data. The previously conceived discrepancies between theory and experiment are all resolved consistently. It is concluded that the computational approach provides a natural way for the understanding of single molecular spectroscopy experiments. The recently proposed non-radiative deactivation mechanism, however, appears to be incoherent when the three cases of YG, WG and GW are considered together.

### Computational methods

The low-energy structures of YG, WG and GW were determined by optimizing a large set of trial structures. The trial structures were generated by considering all possible combinations of the bond rotational degrees of freedom that are illustrated in Fig. 3S of SI. Such a thorough conformational search can in principle provide a complete set of all structures^29–32^. The trial structures were optimized at the M062X/6-31+G** level of theory. Vibrational frequencies were also computed at the M062X/6-31+G** level for all conformers that are within a range of 8 kcal/mol from the global minimum. All the conformers were verified to be the true local minima by the frequency analysis. The single-point energy calculations were carried out using the functional DSD-PBEP86-D3BJ with the basis set of aug-cc-pVTZ. The functional M062X is used here as it is capable of describing H-bonds well at a reasonable calculation cost^20,24,33^. The functional DSD-PBEP86-D3BJ is known to describe H-bond systems with accuracy close to that of CCSD(T) and is used here to provide high quality conformational energies^34,35^.

Based on the DSD-PBEP86-D3BJ/aug-cc-pVTZ energies and the free energy corrections obtained at the M062X/6-31+G** level, the equilibrium Boltzmann distributions of conformers at different temperatures were computed. Conformers of interest were then determined. A conformer of interest here means that it is one of the ten lowest electronic energy conformers, or that its equilibrium concentration is over 1% for some temperature under 450 K.

The structural characteristics of the conformers of interest were analyzed and classified into structural types based on their hydrogen bond (H-bond) features and secondary structures according to the method of Csaszar and Perczel^36^. The transition states between conformers of the same structural type and between representative conformers of different structural types were determined at the DSD-PBEP86-D3BJ/aug-cc-pVTZ//M062X/6-311++G** level using the Berny algorithm^37^.

Computations of the DSD-PBEP86-D3BJ/aug-cc-pVTZ energy were carried out using the ORCA 4.0 software^38^. All the other calculations were performed with the GAUSSIAN 09 suite of programs^39^.

## Electronic supplementary material

Supplementary Information
