# Supplementary material for: Computational study on single molecular spectroscopy of tyrosin-glycine, tryptophane-glycine and glycine-tryptophane
Source: Sci Rep. 2017 Nov 20;7:15869. doi: 10.1038/s41598-017-16234-3 (PMC5696477; doi:10.1038/s41598-017-16234-3)
Supplement: Supplementary file 1 — Supplementary Information [file 41598_2017_16234_MOESM1_ESM.doc]

**Computational study on single molecular spectroscopy of tyrosin-glycine, tryptophane-glycine and glycine-tryptophane**

*Bing Yang^1^, Shixue Liu^2^, Zijing Lin^1^,**

*^1^*Hefei National Laboratory for Physical Sciences at Microscale & CAS Key Laboratory of Strongly-Coupled Quantum Matter Physics, Department of Physics, University of Science and Technology of China, Hefei 230026, China

*^2^*Department of Nanotechnology for Sustainable Energy, Kwansei Gakuin University, Gakuen 2-1, Sanda, Hyogo 669-1337, Japan

^*^ Corresponding author. Tel: +86-551-63606345, Fax: +86-551-63606348, E-mail: zjlin@ustc.edu.cn

**Table 1S:** Relative electronic energies (Energy, in kcal/mol), H-bond networks and structural types (Type) for all WG conformers (Conf.) of interest (W=Tryptophan, G=glycine). The equilibrium distributions (%) at three representative temperatures are also shown but an equilibrium content below 1% is denoted as "-" in most cases.

| Conf. | Energy | H-bonds ^1^* | | | Type ^2^* | Distributions | | |
| --- | --- | --- | --- | --- | --- | --- | --- | --- |
|  |  | Backbone | Main/Side-Chain | |  | 98K | 298K | 450K |
| wg1 | 0.000 | N_PB_H···N_1_; O_T_H···OC_PB_ | N_1_H···π | A1-γ_D_(F)-g+/+ | | 100 | 31.99 | 9.85 |
| wg2 | 0.107 | N_1_H···OC_PB_; O_T_H···OC_PB_ | N_PB_H···π | B-γ_L_(F)-a/- | | - | 7.96 | 2.74 |
| wg3 | 0.733 | N_1_H···OC_PB_; O_T_H···OC_PB_ | N_PB_H···π | A2-γ_D_(F)-g+/+ | | - | - | - |
| wg4 | 1.050 | N_1_H···OC_PB_; O_T_H···OC_PB_ | N_PB_H···π | B-γ_L_(F)-a/- | | - | 2.85 | 1.55 |
| wg5 | 1.118 | N_PB_H···N_1_; O_T_H···OC_PB_ | N_1_H···π | A1-γ_L_(F)-g+/- | | - | 3.73 | 2.25 |
| wg6 | 1.456 | N_PB_H···N_1_; O_T_H···OC_PB_ | N_1_H···π | A1-γ_L_(F)-g+/+ | | - | 4.30 | 3.07 |
| wg7 | 1.484 | N_PB_H···N_1_ | N_1_H···π | A1-ε_D_(E)-g+/- | | - | 1.04 | 0.68 |
| wg8 | 1.498 | N_PB_H···N_1_ | - | A2-ε_D_(E)-g+/- | | - | 1.30 | 0.87 |
| wg9 | 1.537 | N_1_H···OC_PB_ | - | B-β(E)-a/+ | | - | 1.35 | - |
| wg10 | 1.808 | N_PB_H···N_1_; O_T_H···OC_PB_ | - | A2-γ_L_(F)-g+/- | | - | - | - |
| wg11 | 1.809 | N_PB_H···N_1_ | N_1_H···π | A1-ε_D_(E)-g+/+ | | - | 4.63 | 4.00 |
| wg16 | 2.176 | N_PB_H···N_1_; O_T_H···OC_PB_ | N_1_H···π | A2-γ_D_(F)-g-/+ | | - | 1.35 | 1.62 |
| wg19 | 2.280 | N_PB_H···N_1_; O_T_H···OC_PB_ | N_1_H···π | A2-γ_L_(F)-g-/+ | | - | 1.31 | 1.64 |
| wg20 | 2.312 | N_PB_H···N_1_ | N_1_H···π | A1-β(E)-g+/- | | - | 6.24 | 6.60 |
| wg23 | 2.710 | N_PB_H···N_1_ | N_1_H···π | A1-β(E)-g+/+ | | - | 7.52 | 9.00 |
| wg25 | 2.789 | N_PB_H···N_1_; O_T_H···OC_PB_ | N_1_H···π | A2-γ_L_(F)-g-/- | | - | - | 1.04 |
| wg26 | 2.804 | N_PB_H···N_1_; O_T_H···OC_PB_ | N_1_H···π | A2-γ_D_(F)-g-/- | | - | 1.09 | 1.58 |
| wg27 | 3.011 | N_PB_H···N_1_ | N_1_H···π | A1-ε_L_(E)-g+/+ | | - | 1.44 | 2.37 |
| wg30 | 3.208 | N_PB_H···N_1_ | N_1_H···π | A1-ε_L_(E)-g+/- | | - | 1.01 | 1.83 |
| wg31 | 3.279 | N_PB_H···N_1_ | N_1_H···π | A2-ε_D_(E)-g-/+ | | - | 2.87 | 5.32 |
| wg33 | 3.302 | N_PB_H···N_1_; O_T_H···OC_PB_ | N_1_H···π | A1-γ_D_(F)-g-/+ | | - | - | 1.04 |
| wg35 | 3.384 | N_PB_H···N_1_ | N_1_H···π | A2-β(E)-g-/+ | | - | 2.35 | 5.14 |
| wg36 | 3.480 | N_PB_H···N_1_ | N_1_H···π | A2-ε_L_(E)-g-/+ | | - | 1.46 | 3.29 |
| wg40 | 3.601 | N_1_H···OC_PB_ | N_PB_H···π | B-β(E)-a/- | | - | - | 1.93 |
| wg43 | 3.821 | N_PB_H···N_1_ | N_1_H···π | A2-ε_D_(E)-g-/- | | - | - | 1.20 |
| wg46 | 3.915 | N_PB_H···N_1_ | N_1_H···π | A2-β(E)-g-/- | | - | - | 2.63 |
| wg48 | 3.948 | N_PB_H···N_1_ | N_1_H···π | A2-ε_L_(E)-g-/- | | - | - | 1.19 |
| wg56 | 4.361 | N_PB_H···N_1_ | N_1_H···π | A1-ε_D_(E)-g-/+ | | - | - | 1.24 |
| wg60 | 4.487 | N_PB_H···N_1_ | N_1_H···π | A1-β(E)-g-/+ | | - | - | 1.14 |

1* and 2*: See the footnote of Table 1.

**Table 2S:** Relative electronic energies (Energy, in kcal/mol), H-bond networks and structural types (Type) for all GW conformers (Conf.) of interest. The equilibrium distributions (%) at three representative temperatures are also shown but an equilibrium content below 1% is denoted as "-".

| Conf. | Energy | H-bonds ^1^* | | Type ^2^* | Distributions | | |
| --- | --- | --- | --- | --- | --- | --- | --- |
|  |  | Backbone | Main/Side-Chain |  | 98K | 298K | 450K |
| gw1 | 0.000 | N_PB_H···N_1_; O_T_H···OC_PB_ | N_1_H···π | A2-γ_D_(F)-g-/- | 100 | 15.76 | 5.38 |
| gw2 | 0.692 | N_1_H···OC_PB_; O_T_H···OC_PB_ | N_PB_H···π | A1-γ_L_(F)-g+/+ | - | 11.95 | 6.78 |
| gw3 | 0.739 | N_PB_H···N_1_ | N_1_H···π | A1-β(E)-g+/+ | - | 35.09 | 18.06 |
| gw4 | 1.073 | N_1_H···OC_PB_; O_T_H···OC_PB_ | N_PB_H···π | A2-γ_L_(F)-g+/+ | - | 11.47 | 7.85 |
| gw5 | 1.263 | N_1_H···OC_PB_; O_T_H···OC_PB_ | N_1_H···π; N_PB_H···π | A1-γ_L_(F)-g-/+ | - | 1.52 | 1.27 |
| gw6 | 1.669 | N_PB_H···N_1_ | N_1_H···π | A1-δ_L_(E)-g+/+ | - | 4.27 | 3.91 |
| gw7 | 1.716 | N_PB_H···N_1_; O_T_H···OC_PB_ | N_1_H···π | A2-γ_D_(F)-g-/+ | - | 1.12 | 1.18 |
| gw8 | 1.923 | N_PB_H···N_1_; O_T_H···OC_PB_ | N_PB_H···π | A3-γ_L_(F)-g+/+ | - | 1.00 | 1.12 |
| gw9 | 2.034 | N_PB_H···N_1_; O_T_H···OC_PB_ | N_1_H···π | A1-γ_L_(F)-g-/- | - | 1.70 | 1.90 |
| gw10 | 2.193 | N_PB_H···N_1_; O_T_H···OC_PB_ | - | A2-γ_D_(F)-g+/+ | - | - | -- |
| gw12 | 2.633 | N_PB_H···N_1_; O_T_H···OC_PB_ | - | A2-γ_L_(F)-g-/+ | - | 3.50 | 5.48 |
| gw13 | 2.874 | N_1_H···OC_PB_; O_T_H···OC_PB_ | N_PB_H···π | B-γ_L_(F)-g+/+ | - | - | 1.39 |
| gw14 | 2.960 | N_PB_H···N_1_; O_T_H···OC_PB_ | - | A1-γ_D_(F)-g-/+ | - | 2.19 | 4.29 |
| gw16 | 3.231 | N_PB_H···N_1_; O_T_H···OC_PB_ | N_PB_H···π | A2-γ_L_(F)-g+/- | - | - | 1.08 |
| gw17 | 3.294 | N_PB_H···N_1_ | N_1_H···π | A3-β(E)-g+/+ | - | - | 1.53 |
| gw18 | 3.338 | N_PB_H···N_1_; O_T_H···OC_PB_ | - | A2-γ_L_(F)-g-/- | - | - | 1.05 |
| gw19 | 3.368 | N_PB_H···N_1_; O_T_H···OC_PB_ | - | A1-γ_L_(F)-a/- | - | - | 1.30 |
| gw20 | 3.501 | N_1_H···OC_PB_ | - | B-β(E)-g+/+ | - | - | 2.20 |
| gw21 | 3.626 | N_PB_H···N_1_; O_T_H···OC_PB_ | N_PB_H···π | A1-γ_L_(F)-g+/- | - | - | 1.71 |
| gw24 | 3.797 | N_PB_H···N_1_ | - | A2-β(E)-g+/- | - | - | 2.50 |
| gw31 | 4.165 | N_PB_H···N_1_ | - | A1-β(E)-g+/- | - | - | 1.77 |
| gw36 | 4.461 | N_PB_H···N_1_ | N_PB_H···π | A2-α_L_(E)-g+/- | - | - | 1.03 |
| gw44 | 4.810 | N_PB_H···N_1_ | - | B-β(E)-a/+ | - | - | 1.17 |
| gw50 | 4.945 | N_PB_H···N_1_ | - | A2-γ_D_(F)-a/- | - | - | 1.07 |
| gw52 | 5.065 | N_1_H···OC_PB_ | - | B-β(E)-g+/- | - | - | 1.24 |

1* and 2*: See the footnote of Table 1.


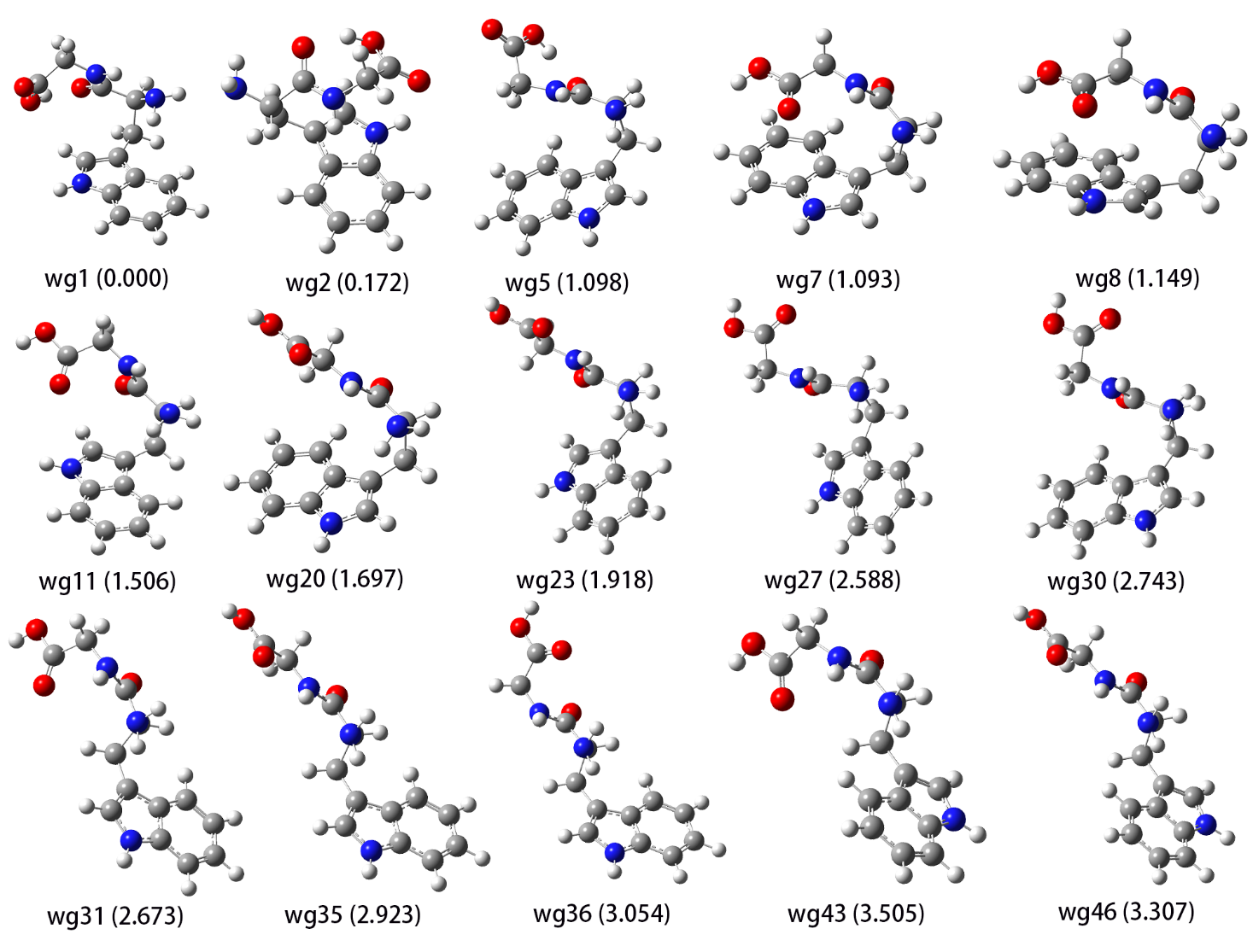


**Figure 1S.** Representative WG conformations. Relative total energies (in kcal/mol) of the conformers are shown in the parentheses.


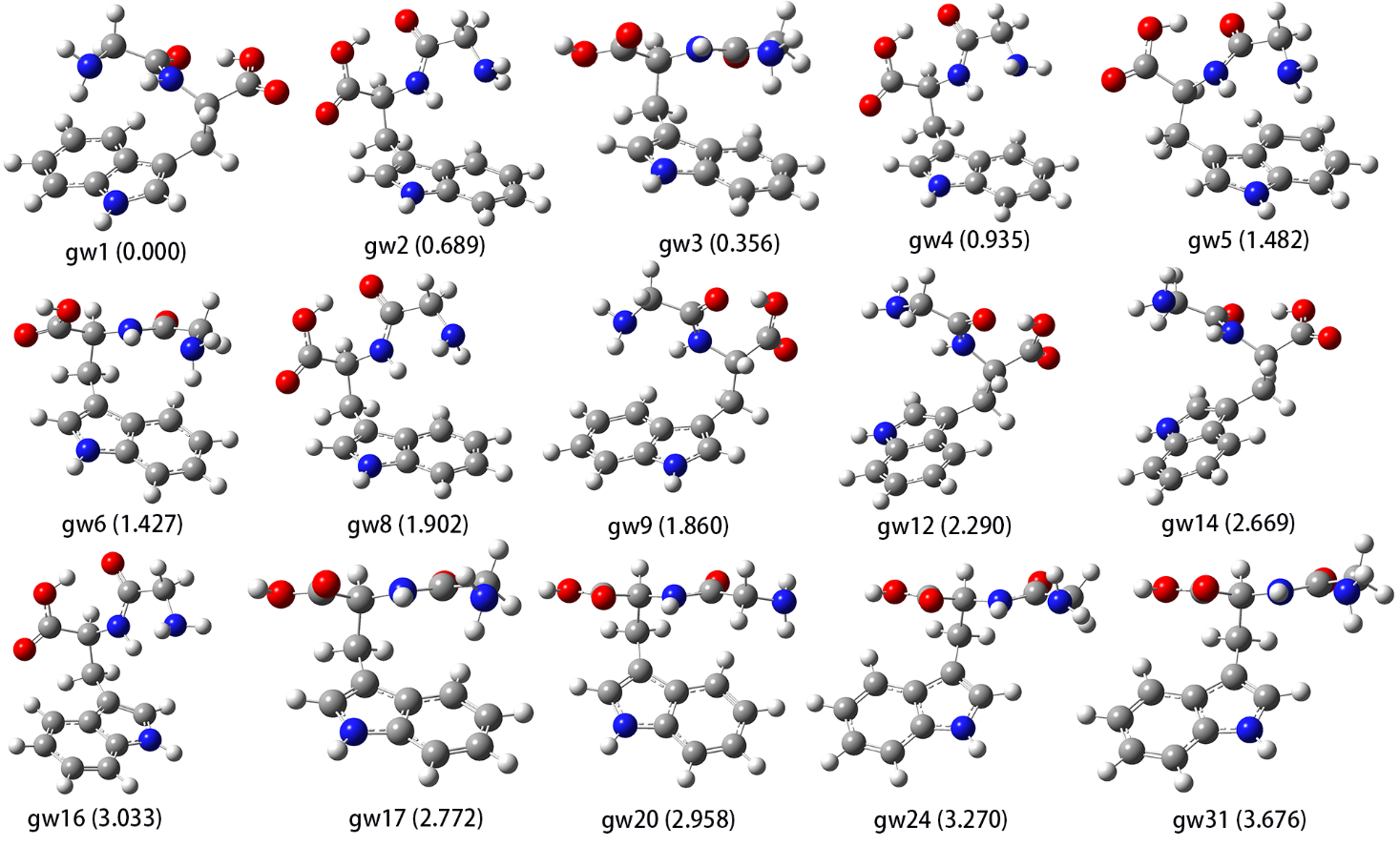


**Figure 2S.** Representative GW conformations. Relative total energies (in kcal/mol) of the conformers are shown in the parentheses.


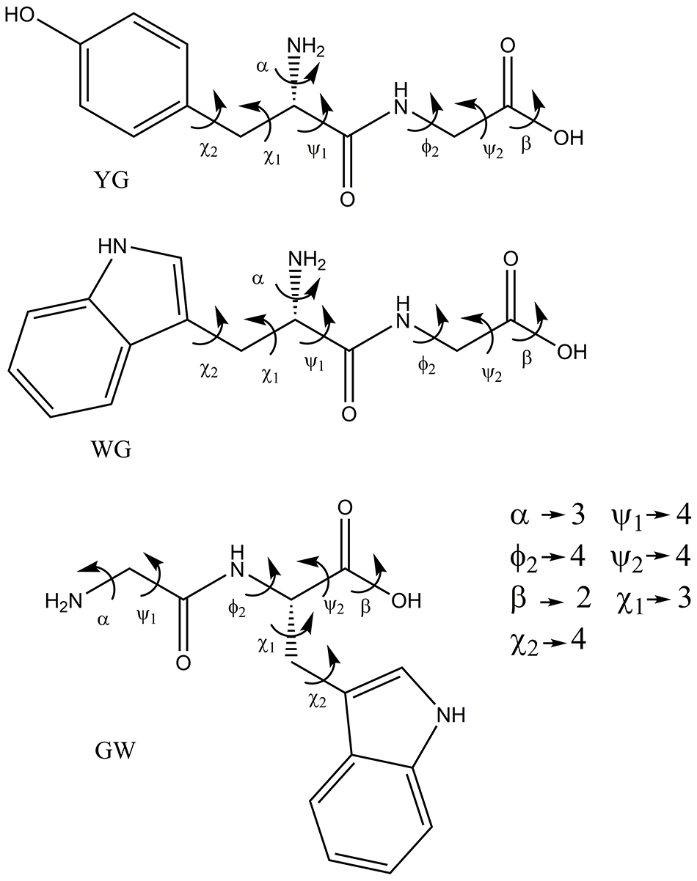


**Figure 3S.** The bond rotational degrees of freedom and the number of rotamers for each degree of freedom in the generation of trial structures for the conformational searches of Tyr-Gly, Trp-Gly and Gly-Trp.
